# Updating the frequency of CCR5Δ32 in Brazil: Descriptive analysis of malaria cases and controls from Acre State, Amazon region

**DOI:** 10.1590/1678-4685-GMB-2025-0056

**Published:** 2026-01-16

**Authors:** Marina Ziliotto, Joel Henrique Ellwanger, Jamille Gregório Dombrowski, Claudio Romero Farias Marinho, Alessandra Pontillo, José Artur Bogo Chies

**Affiliations:** 1 Universidade Federal do Rio Grande do Sul (UFRGS), Departamento de Genética, Laboratório de Imunobiologia e Imunogenética, Programa de Pós-Graduação em Genética e Biologia Molecular (PPGBM), Porto Alegre, RS, Brazil.; 2 Universidade de São Paulo (USP), Instituto de Ciências Biomédicas (ICB), Departamento de Parasitologia, Laboratório de Imunoparasitologia Experimental, São Paulo, SP, Brazil.; 3 Universidade de São Paulo (USP), Instituto de Ciências Biomédicas (ICB), Departamento de Imunologia, Laboratório de Imunogenética, São Paulo, SP, Brazil.

**Keywords:** Amazon forest, Brazil, CCR5, genetics, parasitology

## Abstract

This study updates data on the distribution of CCR5Δ32 allele frequency in populations from Brazilian states. CCR5Δ32 is a variant allele of the *CCR5* gene, which prevents (in homozygosity) or reduces (in heterozygosity) the expression of the CCR5 protein on the surface of leukocytes, being an important factor in infectious diseases. Also, this study reports the frequency of CCR5Δ32 in women from the state of Acre, located in the Brazilian Amazon, a region where the Δ32 allele and malaria are observed concurrently. We found allele frequencies of 2.88% in *Plasmodium*-infected women and of 1.04% among uninfected women. The low Δ32 allele frequencies observed in Acre, compared to other Brazilian regions, could be a reflection of the particular ancestry patterns in current Amazonian populations influenced by past European colonization.

The human C-C chemokine receptor type 5 (CCR5) plays a prominent role in controlling inflammatory responses, thus affecting the course of uncomplicated and severe malaria, a disease caused by infection with parasites of the genus *Plasmodium* and characterized by a significant role of inflammation in its clinical manifestations (Ziliotto et al., 2024). The CCR5 protein is involved in the recruitment and activation of inflammatory cells (particularly T-helper cells, monocytes, and macrophages) to inflammation sites (Ellwanger et al., 2020 a ). In the context of malaria, CCR5-mediated inflammatory responses can influence both protective immunity and immunopathology. While adequate CCR5 function may be necessary for effective parasite clearance, excessive CCR5-mediated inflammation has been associated with severe malaria complications, including cerebral malaria and acute respiratory distress syndrome (Ziliotto *et al.*, 2024).

A 32-base pair deletion in the *CCR5* gene, called CCR5Δ32, prevents (in homozygosity) or reduces (in heterozygosity) the expression of the CCR5 protein on the surface of leukocytes, and therefore this variant may have an influence on CCR5-mediated inflammatory responses observed in parasitic infections (Ellwanger et al., 2020 a ). In this sense, the functional consequences of CCR5Δ32 could potentially alter the balance between protective immunity and harmful inflammation in *Plasmodium*-infected individuals. Despite the role of CCR5 being relevant in malaria-related inflammation and disease outcomes (Belnoue et al., 2003; Cariaco et al., 2023), little is known about the impact of CCR5Δ32 on malaria (Ziliotto et al., 2024). 

The CCR5Δ32 occurs at a frequency that varies from 4% to 6% in most Brazilian states (Kulmann-Leal et al., 2021). However, data on the frequency of CCR5Δ32 in states of the Brazilian Amazon region, where 99% of malaria cases in Brazil occur (Garcia et al., 2022), is scarce (Kulmann-Leal *et al.*, 2021). Given the poor understanding of the influence of CCR5Δ32 on malaria and the limited data regarding the frequency of this variant in the Amazon region, this study aimed to investigate the potential impacts of CCR5Δ32 on malaria in women from the state of Acre, located in the Brazilian Amazon. This study also aimed to update data on the frequency of CCR5Δ32 in Brazilian states.

The sample of this study is composed of 557 pregnant women who were enrolled through volunteer sampling between January 2013 and April 2015, including 365 women infected with *P. falciparum*, *P. vivax*, or both (malaria group) and 192 uninfected women (control group), until delivery. The sample collection aimed to evaluate malaria pathogenesis during pregnancy in Juruá Valley (Acre, Brazil), a municipality that is considered a high-risk area for *Plasmodium* transmission (Dombrowski et al., 2021). Participants received standard antenatal care plus additional monitoring through trained nurse visits during the second and third trimesters to assess clinical outcomes. Additional data on clinical, immunological and demographic characteristics of these women were detailed in previous studies (Dombrowski *et al.*, 2019; Dombrowski *et al.*, 2021). All individuals agreed to participate in this study voluntarily and signed a consent form. This study was approved by the ethics committees of University of São Paulo (CAAE: 32707720.0.0000.5467).

The CCR5Δ32 (rs333) was genotyped by conventional PCR using the following primers: CCR5a 5’-GGTCTTCAT TACACCTGC-3’ and CCR5b 5’-AGGATTCCCGAGTAGCAGATG-3’. The original methodology of this PCR reaction was described by Chies and Hutz (2003). Details of the reaction, with minor adaptations from the original source, are described in Ellwanger et al. (2020 b ).

Firstly, genotype frequencies were compared between the groups. Given that only one individual exhibited the homozygous CCR5Δ32 genotype, individuals were subsequently classified as either “CCR5Δ32 allele carriers” or “CCR5Δ32 allele non-carriers”. Fisher’s exact tests were used to compare the groups. Odds ratio and 95% confidence interval were calculated. The Hardy-Weinberg equilibrium was assessed using the chi-square test separately for the control and malaria groups, as well as for all women combined into a single group. The WinPepi 11.65 (Abramson, 2011) was used to perform the analysis. *P*-value <0.05 was considered as statistically significant.

Furthermore, we have updated the information contained in the Brazil’s map of the CCR5Δ32 allele frequencies observed in Brazilian states previously published by Kulmann-Leal et al. (2021). To minimize bias, only data on the frequency of CCR5Δ32 in control individuals were collected from studies involving Brazilian populations. In addition to the references used by Kulmann-Leal *et al.* (2021) to create the original map (Carvalho et al., 2004; Hünemeier et al., 2005; Ferreira-Fernandes et al., 2015; Silva-Carvalho et al., 2016), we identified in the literature the study by Araujo et al. (2023), which provided new data on CCR5Δ32 frequency for the state of São Paulo, and the study by Lima et al. (2023), which provided new data for the state of Pará. Data on CCR5Δ32 allele frequency for the state of Acre was obtained from this present study, specifically from the control group. To the best of our knowledge, there are no additional data available in the literature on CCR5Δ32 frequency in the other Brazilian States. The map was created with QGIS Desktop 3.28.9 (QGIS, 2025), using geoscience information of the national territory from *Instituto Brasileiro de Geografia e Estatística* (IBGE, 2025) and data of CCR5Δ32 allele frequency as described previously. Geographic coordinates were obtained from SIRGAS2000.

The genotype frequencies do not deviate from Hardy-Weinberg equilibrium (*p*>0.05 in all tests). Considering women from both groups together (*n*=557), the CCR5Δ32 allele frequency was 2.24% (Table 1). Table 2 details the frequencies of the CCR5Δ32 allele and genotypes in controls and malaria group. We observed an allele frequency of 1.04% among uninfected individuals, which is lower than the frequency observed in individuals with malaria (2.88%). However, no statistically significant differences (*p*>0.05) were detected based on comparisons of allele and genotype frequencies, a statistical result that we believe is due to the low sample size combined with the low CCR5Δ32 allele frequency. The observed *p*-values in association with the odds ratio (Table 2) highlight the need for larger sample sizes to draw robust conclusions about any potential relationship between CCR5Δ32 and malaria susceptibility. This point is stressed considering the striking difference between the frequencies of the CCR5Δ32 variant when comparing the sample here evaluated and other populations from different Brazilian geographic regions (Vargas et al., 2006).

**Table 1 -:** CCR5Δ32 genotype and allele frequencies in women from Acre state, Brazil.

CCR5Δ32 profile	All women: control and malaria groups together (n=557)
WT/WT genotype, *n* (%)	533 (95.69%)
WT/Δ32 genotype, *n* (%)	23 (4.13%)
Δ32/Δ32 genotype, *n* (%)	1 (0.18%)
CCR5Δ32 allele frequency	0.0224
WT allele frequency	0.9776

*n*, sample number. WT/WT, wild-type homozygous genotype. WT/Δ32, heterozygous genotype. Δ32/Δ32, variant homozygous genotype.

**Table 2 -:** Comparison of CCR5Δ32 allele and genotype frequencies between the groups.

CCR5Δ32 profile	Control group (n=192)	Malaria group (n=365)	Statistics
WT/WT genotype, *n* (%)	188 (97.92%)	345 (94.52%)	Fisher’s *p*=0.1378
WT/Δ32 genotype, *n* (%)	4 (2.08%)	19 (5.21%)	
Δ32/Δ32 genotype, *n* (%)	0 (0.00%)	1 (0.27%)	O.R. (cases:controls): 2.72 [95% C.I. 0.89-11.11], Fisher’s *p*=0.078
CCR5Δ32 allele carrier, *n* (%)	4 (2.08%)	20 (5.48%)	
CCR5Δ32 allele non-carrier, *n* %)	188 (97.92%)	345 (94.52%)	
CCR5Δ32 allele frequency	0.0104	0.0288	-
WT allele frequency	0.9896	0.9712	-

*n*, sample number. WT/WT, wild-type homozygous genotype. WT/Δ32, heterozygous genotype. Δ32/Δ32, variant homozygous genotype. O.R., odds ratio. C.I., confidence interval.

Figure 1 updates the allele frequency of CCR5Δ32 in Brazilian states. As previously highlighted, CCR5Δ32 occurs at a frequency that varies from 4% to 6% in most Brazilian states (Kulmann-Leal et al., 2021). It is important to point out that the Brazilian population is highly admixed (Kehdy et al., 2015; Kulmann-Leal *et al.*, 2021; Nunes et al., 2025). Historically, it is possible to define parental contributions to the Brazilian population as including, besides the local Amerindian populations, a significant influx of Portuguese individuals, 4 million Africans (mainly from West-Central Africa) and 3.9 million Europeans (other than Portuguese), who arrived in Brazil between the 19th and 20th centuries (Callegari-Jacques et al., 2003). Nevertheless, the distribution of these immigrants was unequal in the various Brazilian regions. Therefore, a low frequency of the CCR5Δ32 in native Amazonian (Hünemeier et al., 2005) and African populations (Kulmann-Leal *et al.*, 2021), as well as particular parental contribution patterns occurring in the Amazonian region, probably reflected in the observed low frequency of such gene variant in our studied sample from Acre.

**Figure 1 -:**
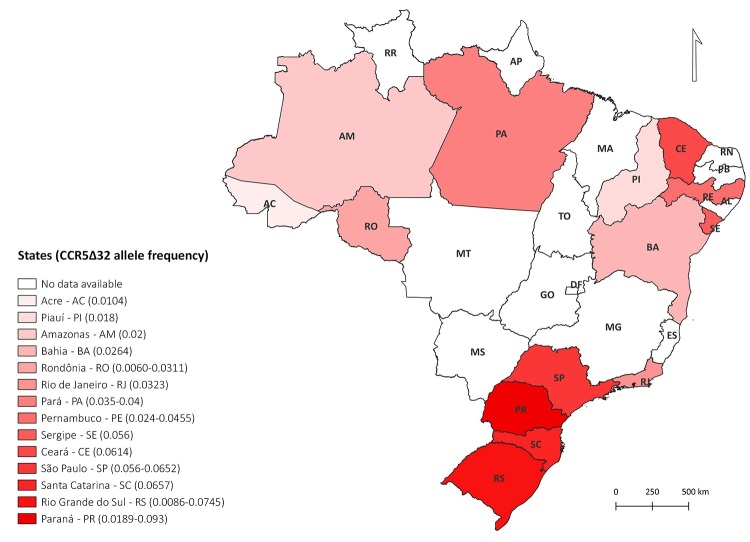
Map of CCR5Δ32 allele frequencies in Brazil. The values in parentheses represents either the single allelic frequency or the lowest and highest allelic frequencies observed in each state. Map adapted from Kulmann-Leal et al. (2021) with additional data from Araujo et al. (2023) and Lima et al. (2023). The graduated map was created using the highest allele frequency value for each state. AC: Acre. AL: Alagoas. AP: Amapá. AM: Amazonas. BA: Bahia. CE: Ceará. DF: Distrito Federal. ES: Espírito Santo. GO: Goiás. MA: Maranhão. MT: Mato Grosso. MS: Mato Grosso do Sul. MG: Minas Gerais. PA: Pará. PB: Paraíba. PR: Paraná. PE: Pernambuco. PI: Piauí. RJ: Rio de Janeiro. RN: Rio Grande do Norte. RS: Rio Grande do Sul. RO: Rondônia. RR: Roraima. SC: Santa Catarina. SP: São Paulo. SE: Sergipe. TO: Tocantins.

The observed difference in CCR5Δ32 frequencies between infected and uninfected women enrolled in our study, although not statistically significant, deserves careful consideration in light of CCR5’s role in malaria-related inflammatory responses. The higher allele frequency in *Plasmodium*-infected individuals (2.88%) compared to controls (1.04%) could reflect the complex role of CCR5 in balancing protective immunity and immunopathology during *Plasmodium* infection. However, it is important to note that without controlling for population stratification, any apparent differences in CCR5Δ32 frequency between cases and controls could also reflect underlying population structure rather than disease association, particularly given the complex admixture patterns characteristic of Brazilian Amazon populations. Given the limited sample size and lack of statistical significance, these findings should be interpreted with caution and require validation in larger studies before drawing conclusions about the potential impact of CCR5Δ32 on malaria susceptibility.

The CCR5Δ32 is observed predominantly in populations from Northern and Eastern European regions, where the variant is considered to be emerged, although today it is reported at very varying frequencies in different populations (Kulmann-Leal et al., 2021). To the best of our knowledge (Kulmann-Leal *et al.*, 2021), this is the first study to describe the frequency of the CCR5Δ32 in the state of Acre, and it identifies a characteristic frequency distinct from other Brazilian regions. It is worth noting that comparisons across different studies may be influenced by variations in study methodologies and population characteristics. However, this study highlights that migration and admixture processes from Europe have left a lasting genetic impact on the Brazilian population, even in remote regions such as the Amazon. 

It is important to acknowledge limitations that should be considered when interpreting the results of the present study. The sample consisted exclusively of women, which reduces the representativeness of the findings for the general population. Although sex-linked differences in CCR5Δ32 frequency are not expected given that the *CCR5* gene is located on chromosome 3 (Ellwanger et al., 2020 a ), an autosomal chromosome, the inclusion of only women limits the generalizability of our findings to the entire population of the state of Acre and the broader Amazon region. Additionally, considering that the frequency of CCR5Δ32 is strongly associated with European ancestry, the lack of control for genetic ancestry may compromise the interpretation of the population data. Future studies should include both sexes and incorporate genetic ancestry analysis to provide a more comprehensive understanding of CCR5Δ32 distribution in Amazonian populations and its relationship with population admixture patterns. 

In conclusion, this study provides descriptive data on CCR5Δ32 frequency in women from the state of Acre, Brazil. Our findings contribute to the broader understanding of genetic diversity of the Brazilian population, particularly from an underrepresented Amazon region in many genetic studies. However, further research with larger and more diverse samples is needed to establish any potential clinical or genetic associations between CCR5Δ32 and malaria. Finally, the differences observed on the frequency of the CCR5Δ32 in the state of Acre, as compared to other Brazilian regions, stresses the need to be quite careful when choosing a control population in genetic association studies.

## Data Availability

 The anonymized data supporting the results of this study are available upon request from the corresponding author.
